# Elastofibrome à double localisation dorsale et thoracique antérieure chez l'enfant

**DOI:** 10.11604/pamj.2015.20.337.1529

**Published:** 2015-04-08

**Authors:** Abdelhalim Mahmoudi, Mouhcine Bendahou, lamiae Chater, Afaf Amarti, Youssef Bouabdallah, My Abderrahmane Afifi

**Affiliations:** 1 Service de Chirurgie Pédiatrique, CHU Hassan II, Fès, Maroc; 2 Service d'Anatomie Pathologique, CHU Hassan II, Fès, Maroc

**Keywords:** Elastofibrome, tumeur, parties molles, traitement, Elastofibroma, tumor, soft tissue, treatment

## Abstract

L’élastofibrome est une pseudotumeur des tissus mous typiquement localisée sous la pointe de l'omoplate. Il est caractérisé par la prolifération de tissus fibreux et adipeux et affecte plus fréquemment les femmes âgées. Nous présentons un cas d’élastofibrome chez une fille de 9 ans.

## Introduction

L’élastofibrome (EF) est une tumeur bénigne, rare et peu connue, décrite pour la première fois par Järvi et Saxen en 1959 [1]. Elle siège essentiellement au niveau de la paroi thoracique postérieure, habituellement au niveau des tissus mous périscapulaires [2]. Nous rapportons l'observation d'une patiente de 9 ans qui présentait un EF dorsal ainsi qu'une localisation basithoracique antérieure droite. L'exérèse chirurgicale a été décidée devant une masse d’évolution chronique et le gène esthétique.

## Patient et observation

Il s'agit d'une fille âgée de 9 ans, sans antécédents particuliers qui s'est présentée pour une tuméfaction de la région dorsale para rachidienne gauche juxtascapulaire et une autre basithoracique droite antérieure évoluant depuis 6 mois, augmentant progressivement de volume. L'examen mettait en évidence une tuméfaction dorsale bien limitée de 5 cm de grand axe, et celle basithoracique de 3 cm, de consistance ferme, non douloureuses à la palpation, mobiles par rapport au plan superficiel et profond. Aucune adénopathie n’était perçue. L’état général était conservé; le bilan biologique était normal.

**Figure 1:**
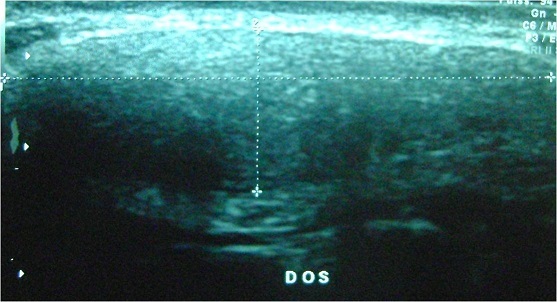
Formations d’échostructure tissulaire hyper-échogene bien limitée siégeant en juxtascapulaire mesurant 5x2cm

**Figure 2:**
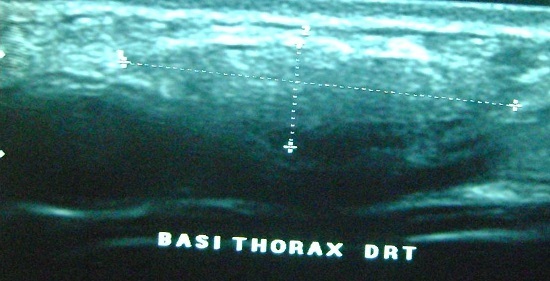
Formations d’échostructure tissulaire hyper-échogenes bien limitées siégeant en basithoracique droite mesurant 3x1cm

**Figure 3:**
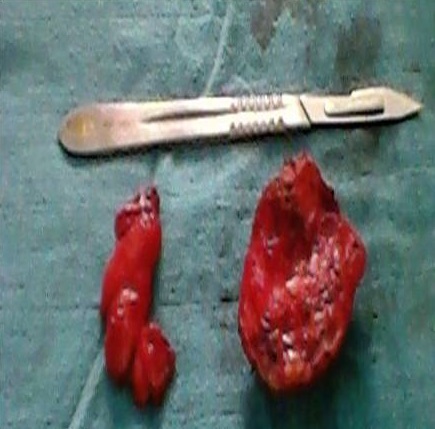
Pieces d'exérèse: aspect macroscopique: tumeurs bien limitées lisses encapsulées

**Figure 4:**
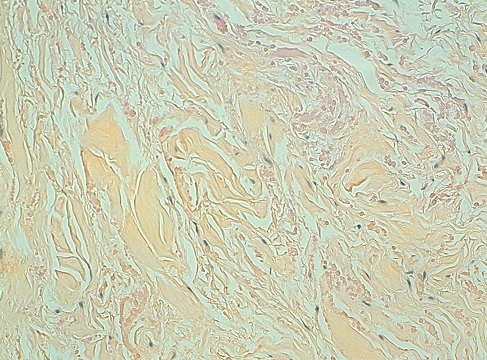
Aspect histologique: HESx200; il s'agit d'une formation à double composante faisceaux de collagène épais mêlés à des fibres éosinophiles correspondant aux fibres élastiques

L’échographie des 2 masses avait montré des formations l'une dorsale et l autre basithoracique antérieure droite, d’échostructure tissulaire, hyperécho gènes bien limitées (Figure 1), présentant un faible signal au Doppler couleur et mesurant respectivement 5x2cm et 3x1 cm (Figure 1, Figure 2). La fille a été opéré devant la gêne esthétique, Les 2 tumeurs ont été emportées en masse (Figure 3). L’étude histologique des pièces d'exérèse objective la présence de fibres de collagènes abondants associés à des fibres élastiques altérés et quelques cellules adipeuses matures permettant de retenir le diagnostic d'un elastofibrome (Figure 4). Les suites opératoires étaient simples avec un recul de 3 ans sans récidive.

## Discussion

L'EF est une tumeur bénigne, rare et d’évolution lente. Elle survient chez 2% des personnes âgées de plus de 60 ans [3] avec une prédominance féminine. L'atteint de l'enfant est extrêmement rare. La pathogénie de l’élastofibrome reste controversée; il serait dû à une réaction hyperplasique, induite par des microtraumatismes répétés entre la scapula et la paroi thoracique, avec dégénérescence de fibres collagènes et production excessive d'un tissu élastique immature par des fibroblastes, ce qui explique le développement plus important des masses du côté droit et leur fréquence chez les travailleurs manuels. Elle siège le plus souvent au niveau de la région infra- et périscapulaire et presque exclusivement adjacente à l'angle caudal de la scapula [2, 4, 5]. Cependant, d'autres localisations ont été rapportées [2]; olécranienne, ischiatiques, interdigitoplantaires, digitales, deltoïdienne, axillaire, trochantérienne…

Sur le plan clinique, la lésion est asymptomatique dans la moitié des cas. Une symptomatologie douloureuse périscapulaire n'est observée, que dans 10% des cas. La localisation bilatérale de l'EF dorsal est relativement peu fréquente avec, en général, un développement asynchrone. Deux localisations différentes peuvent aussi être observées chez un même patient [4, 5]. Comme l'illustre notre observation. L'examen met en évidence une masse ferme, fixée aux plans profonds, mobile par rapport aux plans superficiels et sans signes d'infiltration cutanée. Elle est le plus souvent indolore, mieux palpable. L’échographie est souvent l'examen réalisé de première intention mais non spécifique. Elle montre souvent une masse d’échostructure stratifiée avec alternance de stries hyper et hypoéchogènes parallèles à la paroi thoracique, en regard de la pointe de l'omoplate. Le caractère bilatéral de la masse doit évoquer le diagnostic [6, 7].

La TDM met en évidence une masse de densité équivalente à celle des tissus mous avoisinants, avec des zones de moindre densité [8]. L'IRM montre une lésion souvent hétérogène, bien définie, révélant deux signaux différents en pondération T1. En T2, on observe une augmentation de l'intensité du signal. L'injection du gadolinium ne rehausse pas le signal [8]. Certains articles ont fait mention de la découverte fortuite d'un EF au cours d'une tomographie par émission de positons (TEP scan) [9, 10]. En TEP, l'EF entraîne une captation diffuse et de faible intensité du 18 FDG, traduisant une entité bénigne à faible activité métabolique.

Le traitement des formes symptomatiques est l'exérèse chirurgicale marginale. Pour certains auteurs, même en absence de manifestation clinique lorsque le diamètre est supérieur à 5 cm, il conviendrait de réaliser une résection chirurgicale. Pour d'autres, seule la biopsie de confirmation du diagnostic s'impose en l'absence de symptomatologie [2, 3, 5, 11]. La récidive tumorale locale est très rare et constatée après résection incomplète. Aucun cas de dégénérescence maligne n'est décrit dans la littérature [12, 13].

## Conclusion

L'EF est certes une tumeur rare, voire exceptionnelle chez l'enfant. Son diagnostic est histologique. L'exérèse chirurgicale constitue le traitement des tumeurs symptomatiques, pratiquement sans risque de récidive.
